# The medium helps the message: Early sensitivity to auditory fluency in children’s endorsement of statements

**DOI:** 10.3389/fpsyg.2014.01412

**Published:** 2014-12-04

**Authors:** Stéphane Bernard, Joëlle Proust, Fabrice Clément

**Affiliations:** ^1^Cognitive Science CentreNeuchâtel, Switzerland; ^2^Institut Jean Nicod, École Normale SupérieureParis, France

**Keywords:** testimony selection, trust, fluency, cognitive development, social learning, preschoolers

## Abstract

Recently, a growing number of studies have investigated the cues used by children to selectively accept testimony. In parallel, several studies with adults have shown that the fluency with which information is provided influences message evaluation: adults evaluate fluent information as more credible than dysfluent information. It is therefore plausible that the fluency of a message could also influence children’s endorsement of statements. Three experiments were designed to test this hypothesis with 3- to 5-year-olds where the auditory fluency of a message was manipulated by adding different levels of noise to recorded statements. The results show that 4 and 5-year-old children, but not 3-year-olds, are more likely to endorse a fluent statement than a dysfluent one. The present study constitutes a first attempt to show that fluency, i.e., ease of processing, is recruited as a cue to guide epistemic decision in children. An interpretation of the age difference based on the way cues are processed by younger children is suggested.

## INTRODUCTION

Recently a growing number of studies in developmental psychology have underlined the importance of testimony in knowledge acquisition (e.g., Clément, 2010; Harris, 2012). Given the risk of being misguided by manipulative or misinformed sources (Sperber et al., 2010), one of the objectives of this line of research is to determine whether children are able to assess the comparative credibility of information provided by a speaker. A typical paradigm for testing endorsement of statements or of object labels involves contrasting two sources stating contradictory propositions and asking children to choose between the two statements (the conflicting sources task). So far, the main focus has been on properties of the source of the testimony, such as reliability (e.g., Koenig and Harris, 2005; Pasquini et al., 2007; Birch et al., 2008; Scofield and Behrend, 2008; Corriveau and Harris, 2009a), expertise (Sobel and Corriveau, 2010; Koenig and Jaswal, 2011) or the emotions displayed by informants (Clément et al., 2013). Little is known, however, about the influence of the message itself on testimony selection. Bernard et al. (2012) asked whether children would more readily follow an informant using the connective *because* to link an argument in a statement than an informant giving very similar information without the connective. They showed that while 4- and 5-year-olds used the connective *because* to guide their selective trust, 3-year-olds demonstrated indiscriminate selective trust. The same authors have recently shown that 3-year-olds could take into account certain informational properties of a message in selecting testimonies (Mercier et al., 2014). In fact, 3- to 5-year-olds preferred to endorse statements including a non-circular argument over a circular one (see also Corriveau and Kurkul, 2014, and for older children: Baum et al., 2008). The three experiments reported in this paper explore a cue embedded in the message that has not been yet investigated: the fluency of the communicated information.

Processing fluency can be defined as “the ease or difficulty with which new, external information can be processed” (Schwarz, 2004, p. 338). Fluency, or ease of processing, is what a person may experience when attempting to perceptually discriminate objects with a given property (perceptual fluency), retrieve items from episodic or semantic memory (retrieval fluency), or understand the communicated content (conceptual fluency; see Alter and Oppenheimer, 2009). Dysfluent information is information that is more difficult to process, requiring, for instance, a longer latency before any decision can be reached by adults (see Koch and Forgas, 2012). Several studies on adults support the prediction that fluent statements are more readily endorsed than dysfluent ones: fluent stimuli subjectively appear to be more credible, and are more frequently judged as probably true on a Likert scale than dysfluent ones (for a meta-analysis of this effect, see Dechêne et al., 2010). Regarding this effect, fluency has been, for instance, manipulated through repeated exposure to statements, which enhances the ease of processing (Johnston et al., 1985). A number of studies have shown that repeated statements were judged as more credible in comparison to statements that had not been presented before (e.g., Hasher et al., 1977; Begg et al., 1992; Brown and Nix, 1996). The effect of repeated exposure on credibility judgment could be explained as follows: processing a repeated statement is experienced as unexpectedly fluent. This unexpected fluency is experienced as discrepant from a comparison standard. Research has shown that an experienced discrepancy between expected and actual fluency of a stimulus generates feelings of familiarity (e.g., Whittlesea et al., 1990; Whittlesea and Williams, 1998, 2000; Whittlesea, 2004). These feelings involve the sense that something has been encountered before. They are generated by the higher fluency which previous exposure confers to perceptual and memorial processing of a given item. It has been hypothesized that these feelings of familiarity in turn affect credibility judgments because experiencing a stimulus as familiar could lead subjects to feel that they have seen or heard this stimulus before, suggesting that it is probably more credible (e.g., Reber and Schwarz, 1999; Weaver et al., 2007; Schwarz, 2010).

Arguably, any other variable that increases processing fluency might have the same epistemic effect as prior exposure, and in fact people make similar credibility judgments about statements that are fluently processed for other reasons than repetitive presentation. Studies have demonstrated that other instantiations of fluency, such as rhyme in aphorisms (McGlone and Tofighbakhsh, 2000) or high contrast in the visual presentation of statements (Reber and Schwarz, 1999; Koch and Forgas, 2012), also influence credibility judgments. For example, Reber and Schwarz (1999) manipulated the visual fluency of statements: various color fonts were used on a computer screen to make the statements more or less difficult to read against a white background. After each presentation, adult participants were asked if the statement presented (e.g., “Osorno is in Chile”) was true or false. The statements were more likely to be judged as true when presented in a highly visible color, i.e., in a fluent presentation.

The three experiments reported in this paper manipulated the auditory fluency of the message by adding different levels of noise to statements presented to children. Even though the effect of fluency on credibility with this type of auditory manipulation has not been previously tested in adults, research on memory has shown that this kind of manipulation generates different levels of fluency. For instance, in a study by Goldinger et al. (1999), adults were briefly presented with lists of words and the auditory fluency was manipulated by presenting the words with soft or loud background noise. Adults were then presented with a recognition test (recognition judgments). Results indicated that adults were more likely to consider a word as “old” (sense of familiarity) when it was presented with the soft background noise (fluent presentation) than with the loud background noise (dysfluent presentation; for other operationalizations of auditory fluency see Rhodes and Castel, 2009; Besken and Mulligan, 2013). From a more general perspective, it has been shown that fluency influences memory assessment. In particular, it has been established that a wide range of perceptual fluency affects judgments of learning (i.e., people’s estimations of how well they have learned something) and recognition judgments (e.g., Kelley and Rhodes, 2002; Kornell et al., 2011; McDonough and Gallo, 2012; Yue et al., 2013).

Given that adults judge a fluent statement to be more credible than a dysfluent one, one can expect that, in the absence of other cues, children should be more willing to endorse a statement that is easier to process. There are no studies directly addressing this question in the case of preschoolers, and, more generally, nothing is known about the possible influence of fluency on child development. If two conflicting statements, one fluent and the other dysfluent, are presented by two different informants (as in the classical paradigm used in research on testimony with children), children should selectively endorse the more fluent statement. This selectivity would be based only on the fact that higher fluency makes a statement more credible, as in adult studies. In the three experiments described below, the possible role of the auditory fluency of a message on endorsement of statements was investigated. Participants were as young as 3-years-old, an age at which it has been demonstrated that children are able to take part in the conflicting sources task (Koenig et al., 2004), and as old as 5, to see if there were any developmental differences. These possible developmental differences are investigated given that previous studies have reported developmental changes in testimony selection between 3- and 5-year-olds when the message itself was manipulated and not the properties of the informants (Bernard et al., 2012; Corriveau and Kurkul, 2014; Mercier et al., 2014).

## EXPERIMENT 1

### METHOD

#### Participants

This experiment involved 81 children: 27 3-year-olds (11 girls, *M*age = 42.66 months, SD = 3.80, range 36–47 months), 26 4-year-olds (11 girls, *M*age = 52.88 months, SD = 3.69, range 48–59 months), and 28 5-year-olds (15 girls, *M*age = 67.03 months, SD = 3.52, range 61–71 months) from a school in Lyon (France). All the participants were French, and all the experiments were conducted in French. Most children were from middle- and upper-middle-class families. Each child was tested individually in a quiet room by a single experimenter for about 10 min. Informed parental consent was obtained for each child. All participants were treated according to the Declaration of Helsinki.

#### Materials and procedure

**FIGURE 1:**
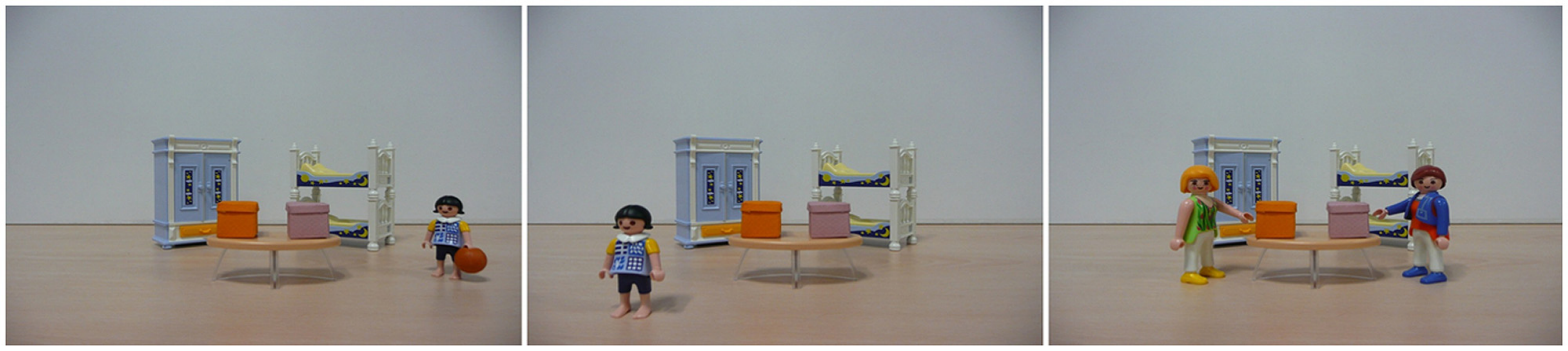
**Vignettes for the ball story**.

To test whether children are more likely to endorse a fluent than a dysfluent statement, a conflicting sources task was used. Three stories were presented to children on a computer screen. All stories were constructed on the same model: in the first vignette, a young Playmobil girl arrived at a place where two closed colored boxes are sitting on a table (places counterbalanced). This girl was carrying an object, and the experimenter explained that the girl was going to put this object in one of the two boxes. A second vignette showed the same girl without the object. The experimenter explained that she was leaving after having put the object in one of the boxes; he specified that he did not know which of the two boxes contained the object. Then, the experimenter showed a third vignette where two new female Playmobil characters were depicted (**Figure 1**).

The experimenter gave the names of the two characters and said that each one of them would help him find the object. Children wore headphones in order to listen to what the characters said.

For the first character, an animation bubble popped up and a voice recording was activated; the child heard, for instance, “The ball is in the orange box.” The same procedure was repeated with the second character, who stated, for instance, “The ball is in the pink box” (the characters’ locations, the order of bubble activation, the fluency of each sentence and the voice attributed to a character were all counterbalanced).

Finally, the experimenter asked the children, “According to you, where is the ball?” If the children did not respond, the experimenter asked: “Is the ball here (pointing at one of the boxes) or is the ball here (pointing at the other)?”

The two other stories were built on the same model. In each of them, the young girl, the object carried, the colors of the boxes and their places (right/left), the two female informants, their names and the voices associated with them were varied. The child could obtain a maximum score of 3 points (1 point for each story when the box linked to the more fluent statement was chosen).

After the three stories, children listened to each statement again to evaluate their comprehension—this was done by asking them to repeat each statement. This step was used to ensure that children’s choices were not linked to any difficulty of comprehension. All children succeeded at this comprehension test.

All sentences were recorded and modified using Audacity software. The sentences were digitized (32-bit resolution) at a 44.1-kHz sampling rate. For each story (*N* = 3), two different volunteers were asked to say the two sentences corresponding to the two locations in a neutral tone.

The sentences were normalized to produce an output level of 80 dB-A (A-weighted decibels: audible decibels measured with an artificial ear) and standardized to 2.1 s in duration. Two separate copies were created for each sentence and modified by adding Brownian noise to obtain two levels of auditory fluency: a fluent statement (amplitude of Brownian noise = 50 dB-A) and a dysfluent statement (amplitude of Brownian noise = 65 dB-A). Brownian noise (or red noise) is the kind of signal noise produced by Brownian motion (random process). The sound is a low roar resembling a waterfall and has a “soft” quality compared to white and pink noise.

To test whether these two levels of fluency were perceptually discriminated, 23 adults were recruited (13 women, *M*age = 23.88 years, SD = 5.5 years, age range 19–44 years). The adults listened to the stimuli and rated the recording quality for each sentence on a scale from 1 (very good quality) to 7 (very bad quality). The results showed that the fluent statements (*M* = 2.39, SD = 0.89) were evaluated as having better auditory quality than the dysfluent statements [*M* = 5.26, SD = 1.17, *t*(44) = 9.32, *p* < 0.001].

### RESULTS

**FIGURE 2:**
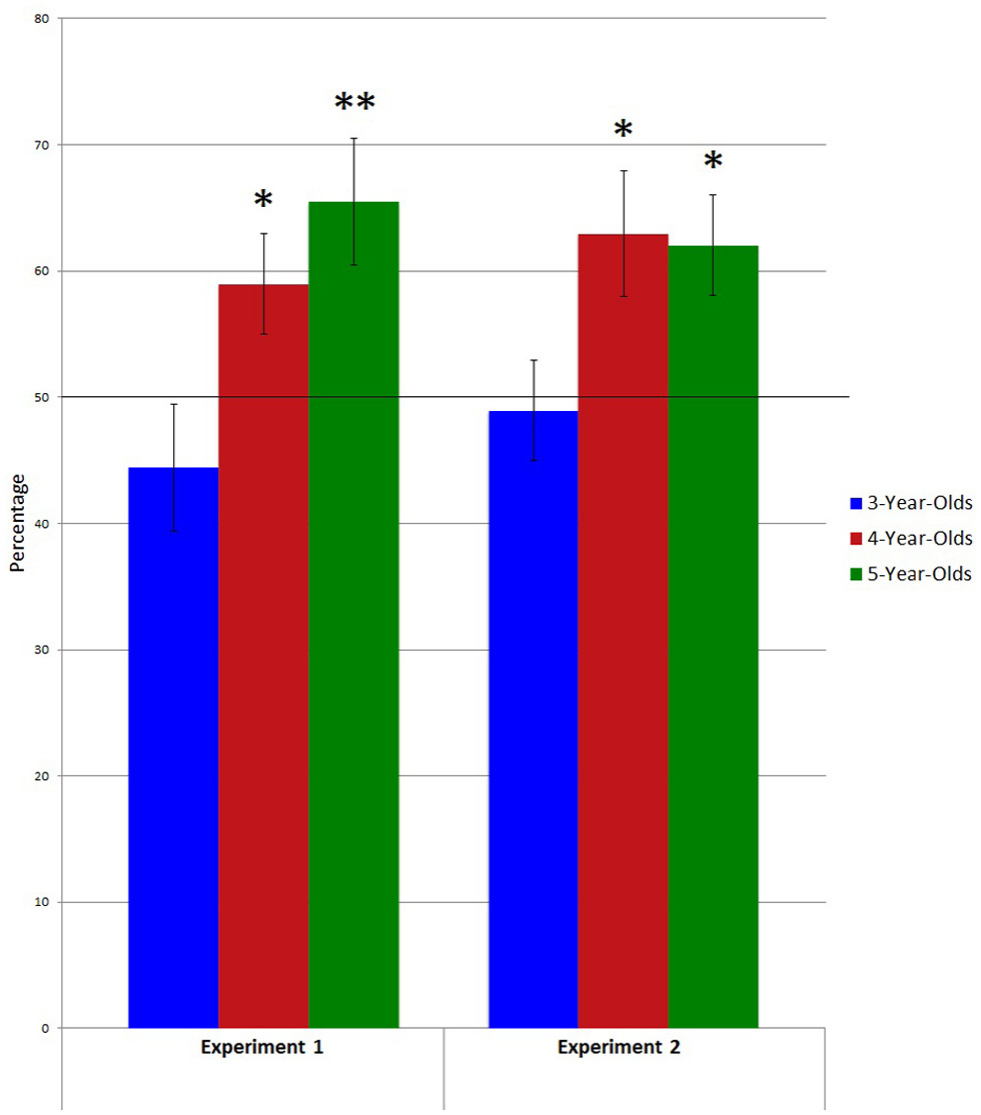
**Percentage of fluent choices in each Experiment and in each age group (the chance line is at 0.50).** *p < 0.05, **p < 0.01, one-sample *t*-test.

The percentage of choices elicited by fluent sentences was 44.4% for the 3-year-olds, 58.9% for the 4-year-olds, and 65.5% for the 5-year-olds (**Figure 2**). A 3 (age group: 3-year-olds, 4-year-olds, 5-year-olds) × 2 (gender: girl, boy) analysis of variance (ANOVA) with the score of fluent choices as a dependent variable yielded a significant main effect only for the age group factor, *F*(2,75) = 4.75, *p* < 0.05. The 5-year-olds chose the fluent statements significantly more^1^ (*M* = 1.96, SD = 0.84) than the 3-year-olds (*M* = 1.33, SD = 0.78). 4-year-olds’ performance (*M* = 1.77, SD = 0.65) was not significantly different from that of the 3-year-olds or from that of the 5-year-olds.

However, children’s choices elicited by fluent sentences were significantly above chance for the 4-year-olds, *t*(25) = 2.11, *p* = 0.04, and 5-year-olds, *t*(27) = 2.93, *p* = 0.01, but not for the 3-year-olds, *t*(26) = -1.10, *p* = 0.28. These findings could be explained by the fact that the ANOVA did not take the chance level into account or test hierarchical relations between means (in this case, the ANOVA tested the null hypothesis *M*1 = *M*2 = *M*3). Following the difference between the groups in terms of chance and the percentages of choices elicited by fluent sentences, another hypothesis can be tested: the 3-year-olds choose the fluent statements less often than the 4-year-olds; and the performance of the 4-year-olds is similar to that of the 5-year-olds. To test this hypothesis more specifically, a contrast analysis was used (see e.g., Wendorf, 2004). This kind of analysis is more powerful for testing specific hierarchical hypotheses than classical analyses, and can be used even if the classical test is not significant (Brauer and McClelland, 2005). Two contrasts were tested in a regression analysis: a contrast of interest^2^, corresponding to the previous hypothesis regarding the age difference, and an orthogonal contrast, which tested the residual variance. The hypothesis can be accepted if the contrast of interest predicts the choices of the fluent statements and if the orthogonal contrast does not. The contrast analysis confirmed this hypothesis: *F*(1,78) = 2.96, *p* = 0.004, for the contrast of interest, and *F*(1,78) = 0.94, *p* = 0.35, for the orthogonal contrast.

### DISCUSSION

This first experiment showed that 4- and 5-year-olds use auditory fluency to select which testimony to endorse. These results are the first to indicate that fluency could play a role in testimony selection by children and, from a wider perspective, could play an epistemic role in child development.

The fact that sensitivity to fluency is only elicited in 4- and 5-year-olds demands an explanation. First, it could be hypothesized that 3-year-olds do not discriminate between fluent and dysfluent sentences. In fact, anatomical data has shown that children do not reach the adult level of myelination for the auditory system until around age 4–5 (Kinney et al., 1988; Moore and Linthicum, 2007). Thus it is likely that 3-year-olds cannot use auditory fluency because their perceptual system does not provide sufficiently contrasted information about the quality of the signals. Given that Experiment 1 tested only how discrepancy in fluency was perceived by adults, we decided to include in a second experiment a measure to evaluate if the two levels of fluency manipulated were perceptually discriminated by 3-, 4-, and 5-year-old children.

The difference between the 3-year-olds’ results and those of older children could also be explained in another way: one could consider that children needed to reason about the knowledge of others in order to succeed in the conflicting sources task of Experiment 1. If this is the case, one should expect that this task required social cognitive abilities such as a theory of mind. Indeed, given that the ability to pass false belief tasks develops between 3 and 4 years of age (Wellman et al., 2001), it is plausible that the difference between 3-year-olds and older children was related to a difference in theory of mind development, even if the relation between theory of mind and testimony selection is a matter of debate (e.g., Proust, 2012; Lucas et al., 2013). In order to test this hypothesis, children were therefore presented with three false belief tasks in Experiment 2.

Finally, regarding the results of the older children, one could argue that the discrepancy in fluency may lead children to attribute characteristics such as familiarity or in-group membership to the informant who used the fluent statement. In fact, recent studies have shown that familiarity and group membership influence testimony selection in young children (e.g., Corriveau and Harris, 2009b; MacDonald et al., 2013). In order to ensure that children’s choices were not linked to a preference for the informant whose message was more fluent, a preference question was also introduced in Experiment 2.

## EXPERIMENT 2

### METHOD

#### Participants

This experiment involved 78 children: 24 3-year-olds (9 girls, *M*age = 42.83 months, SD = 3.69, range 37–47 months), 27 4-year-olds (12 girls, *M*age = 53.33 months, SD = 4.06, range 48–59 months), and 27 5-year-olds (17 girls, *M*age = 67.29 months, SD = 3.41, range 62–71 months) from two schools in Lyon (France). The measure of children’s auditory evaluation of the two levels of fluency involved 66 children: 22 3-year-olds (11 girls, *M*age = 41.5 months, SD = 3.76, range 36–47 months), 23 4-year-olds (17 girls, *M*age = 54.39 months, SD = 3.31, range 49–58 months), and 21 5-year-olds (9 girls, *M*age = 65.33 months, SD = 3.77, range 60–71 months) from a school in Lyon. The demographics were similar to those of Experiment 1. Each child was tested individually in a quiet room by a single experimenter for about 10 min. Informed parental consent was obtained for each child. All participants were treated according to the Declaration of Helsinki.

### MATERIALS AND PROCEDURE

#### Conflicting sources task

In the first vignette, a young Playmobil girl, Pauline, and her dog, were presented to the children on a computer screen. The experimenter explained that Pauline’s dog often escapes and Pauline has to look for him. Then the experimenter said: “In this game, you will try to help Pauline find her dog.”

A second vignette showed Pauline facing two Playmobil female characters. Each one pointed in a different direction. The experimenter said: “One day, Pauline is looking for her dog in a park. These two ladies tell her something. We are going to listen to them.” Using the same headphones as in Experiment 1, the child heard the first character say: “The dog went over there.” The same procedure was repeated with the second character. This second character used the exact same sentence (“The dog went over there”) but pointed in a different direction (the characters’ locations, the order of bubble activation, the fluency of each sentence and the voice attribution for the character were again counterbalanced). The fluency of the messages was manipulated in the same way as in Experiment 1. Finally, the experimenter asked the children, “According to you, where did Pauline’s dog go?”

For each of the three following trials, two new characters pointed to a different location and stated: “The dog went over there.” The background of each picture, the female characters and their voices were varied. The child could obtain a maximum score of 4 points (1 point for each story when the direction provided with the more fluent sentence was chosen — children indicated their choices by pointing to one of the two directions, that is to one side of the screen).

After the four stories, children listened to each statement again to allow evaluation of (1) their comprehension — by asking them to repeat each statement; and (2) their preference for each informant — by asking them after these two statement repetitions following each story: “Do you prefer this lady (pointing) or this lady (pointing)?” The first step was undertaken to ensure that children’s choices were not linked to any difficulty of comprehension. As in Experiment 1, all children succeeded at this comprehension test. The second question was asked in order to ensure that children’s choices were not linked to a preference for the informant whose message was more fluent. Each child could obtain a maximum score of 4 points related to these preference choices (1 point for each story when the character linked to the more fluent statement was chosen).

The fluent and dysfluent statements were created in the same way as in Experiment 1. Since there were four stories, they were recorded this time by eight female volunteers. As the sentences were slightly shorter, they were standardized to a duration of 1.9 s.

#### False belief tasks

In order to evaluate theory of mind abilities, children were presented with three false belief tasks. The first task was an unexpected transfer task adapted from Wimmer and Perner (1983). In this task, a story character put an object in one place and then left. Another character moved the object, putting it in another place and then the first character came back. The child was asked a test question (a belief question): “Where will (story character) look for her (object)?” Two control questions were asked to check that the child remembered both the original place and the actual one. These control questions had to be answered correctly so that credit could be given for the test questions. The test question was allotted a binary score (1/0).

The second task was an unexpected content task adapted from Perner et al. (1987). In this task, the child had to identify the content of a familiar box. After having answered correctly, the child could open the box and see the unexpected content inside. Then the box was closed and the child was asked the following test questions: a “self” belief question, “Before opening the box, what did you think was inside?,” and an “other” belief question about a friend who had not looked inside, “(name of the friend) has not looked inside the box. What will he/she think is inside the box before opening it?” One control question was asked to check whether the child remembered the actual content of the box. As in the first task, this control question had to be answered correctly before credit could be given for the test questions. The test questions were allotted a binary score (1/0).

The third task was another unexpected one, adapted from Jenkins and Astington (1996). In this task, the child was shown a picture book in which a partial view of what seemed to be a rabbit’s tail was in fact a lady’s hairstyle bun (unexpected picture). The child was asked the following test questions: a “self” belief question, “Before I turned the page, what did you think this was?” and an “other” belief question about a friend, “(name of the friend) has not seen this image. Before I turn the page, what will he/she think it is?” The test questions were allotted a binary score (1/0). For these three tasks, the child could therefore obtain a maximum raw score of 5.

#### Auditory evaluation task

**FIGURE 3:**
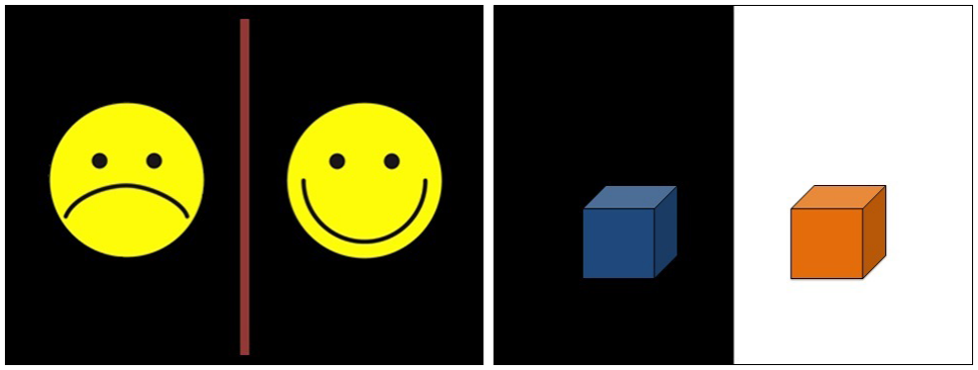
**Excerpt of screen presentation for the auditory evaluation task (left) and for the reaction time task (right)**.

To test if the two levels of fluency were perceptually discriminated by children of each age group, another group of children were asked to evaluate the recording quality of the sentences used in the conflicting sources task of Experiment 2. The task consisted of a warm-up (six items: three fluent, three dysfluent) and then the task *per se* (eight items: four fluent, four dysfluent). For each item, children were shown a picture of two yellow smiley faces (**Figure 3**). The experimenter said to the children: “You are going to hear some girls. These girls are going to say something. They will always say the same thing but sometimes you will hear quite clearly what they say and sometimes you will not hear very clearly what they say. If you hear quite clearly what they say, you have to press this button (the experimenter pointed to the button on the keyboard with the corresponding smiley). If you don’t hear very clearly what they say, you have to press this button (the experimenter pointed to the button on the keyboard with the corresponding smiley).” Using the same headphones as in the conflicting sources task, children then heard a sentence (i.e., “ The dog went over there”) and the experimenter asked: “Did you hear quite clearly what the girl said (the experimenter pointed to the corresponding button) or did you not hear very clearly what the girl said (the experimenter pointed to the corresponding button; order counterbalanced)?” These instances of instruction and feed-back were only used during the warm-up. The task was created using the software program E-prime to randomly present the sentences and to record which button was pressed by the child. For each item, 1 point was attributed to children when they pressed the button “heard quite clearly” and 0 when they pressed the button “not heard very clearly.”

### RESULTS

#### Conflicting sources task

The percentage of choices associated with fluent sentences was 48.9% for the 3-year-olds, 62.9% for the 4-year-olds, and 62.1% for the 5-year-olds (**Figure 2**). An ANOVA failed to reveal any significant main effects or interaction effects between age group and gender factors. However, as in Experiment 1, children’s choices elicited by fluent sentences were significantly above chance for the 4-year-olds, *t*(26) = 2.33, *p* = 0.03, and 5-year-olds, *t*(26) = 2.47, *p* = 0.02, but not for the 3-year-olds, *t*(23) = -0.24, *p* = 0.81. Following these differences between the groups in terms of chance and the percentages of choices elicited by fluent sentences, a contrast analysis was also conducted to test the same hypothesis as in Experiment 1: the 3-year-olds choose the fluent statements less often than the 4-year-olds; and the performance of the 4-year-olds is similar to that of the 5-year-olds. The contrast analysis confirmed this hypothesis: *F*(1,75) = 2.16, *p* = 0.03, for the contrast of interest, and *F*(1,75) = -0.133, *p* = 0.89, for the orthogonal contrast.

#### False belief tasks

Regarding the three false belief tasks, nine children were not able to respond correctly to the control questions: four 3-year-olds, four 4-year-olds and one 5-year-old. They were thus removed from the following analyses. The percentage of success for all the false belief tasks was at 34% for the 3-year-olds, 57.4% for the 4-year-olds, and 83.8% for the 5-year-olds.

These results correspond to the classical trend in the theory of mind literature for this kind of explicit task (e.g., Wellman et al., 2001). Given that this false belief evaluation was conducted to test the relation between fluent choices and theory of mind abilities, the next step in our analysis presents the correlations between false belief scores and fluent choices.

#### Correlations

There were no significant correlations between the fluent choices and the false belief scores, both for all children, *r* = 0.10, *p* = 0.41, and for each age group (3-year-olds: *r* = -0.21, *p* = 0.36; 4-year-olds: *r* = 0.19, *p* = 0.37; 5-year-olds: *r* = -0.08, *p* = 0.68).

To better understand the relation between the fluent choices and the theory of mind abilities, the children who succeeded in the false belief evaluation (i.e., by getting 3 points or more out of the maximum raw score of 5) and those who did not (i.e., who got less than 3 points) were distinguished. The performance of these two groups of children was not significantly different for fluent scores, for all children, *t*(67) = 0.71, *p* = 0.48, and for each age group [3-year-olds: *t*(18) = -0.31, *p* = 0.75; 4-year-olds: *t*(21) = 0.73, *p* = 0.47; 5-year-olds: *t*(24) = -0.85, *p* = 0.40].

#### Preference

A 3 (age group: 3-year-olds, 4-year-olds, 5-year-olds) × 2 (gender: girl, boy) analysis of variance with the score of preference for the informant linked to the fluent statement as a dependent variable yielded no significant main effect or interaction effect between these two factors. For all children, these choices did not significantly differ from chance [*M* = 2.11, SD = 1.06, *t*(77) = 0.95, *p* = 0.34].

#### Auditory evaluation task

A 3-way ANOVA with age group (3-year-olds, 4-year-olds, 5-year-olds) and gender (girl, boy) as between-subjects variables and fluency level (fluent, dysfluent) as within-subjects variable was performed for the scores in auditory evaluation. This revealed only a significant main effect of fluency level, *F*(1,60) = 21.46, *p* < 0.001. The fluent statements (*M* = 2.53, SD = 0.96) were evaluated as having better auditory quality than the dysfluent statements (*M* = 1.68, SD = 0.93, *p* < 0.001).

However, 3-year-old children’s evaluation of fluent sentences (*M* = 2.40, SD = 1.09) was not significantly different from that of dysfluent sentences [*M* = 1.91, SD = 1.19, *t*(21) = 1.53, *p* = 0.14]. Moreover, these two evaluations did not differ significantly from chance [fluent sentences: *t*(21) = 1.74, *p* = 0.10; dysfluent sentences: *t*(21) = -0.35, *p* = 0.72]. In contrast, 4-year-old children’s evaluation of fluent sentences (*M* = 2.74, SD = 1.01) was significantly different from that of dysfluent sentences [*M* = 1.48, SD = 0.85, *t*(22) = 4.57, *p* < 0.001]. These two evaluations differed significantly from chance [fluent sentences: *t*(22) = 3.51, *p* = 0.002; dysfluent sentences: *t*(22) = -2.96, *p* = 0.007]. Finally, 5-year-old children’s evaluation of fluent sentences (*M* = 2.43, SD = 0.75) was significantly different from that of dysfluent sentences [*M* = 1.66, SD = 0.65, *t*(20) = 2.96, *p* = 0.008]. These two evaluations differed significantly from chance [fluent sentences: *t*(20) = 2.63, *p* = 0.016; dysfluent sentences: *t*(20) = -2.32, *p* = 0.031].

### DISCUSSION

The results of Experiment 2 replicate and extend those of Experiment 1. The fluency with which a message is provided influences 4- and 5-year-old children’s endorsements of statements. Moreover, our complementary results enable us to propose an explanation of the age-group differences.

First, given the results regarding the relation between fluent choices and false belief scores, it seems that the 3-year-olds’ results cannot be explained in terms of a theory of mind deficit. A possible explanation for the 3-year-olds’ failure to use fluency might be that they do not yet have a sufficiently fine-tuned sensitivity to the auditory quality of messages to discriminate between the two messages heard. The results of our auditory evaluation task seem to validate this hypothesis. During this task, only 4- and 5-year-old children were able to distinguish fluent and dysfluent sentences in terms of their auditory quality. Moreover, the hypothesis that 3-year-olds do not have a sufficiently fine-tuned sensitivity the auditory quality of messages could be supported by anatomical data (Kinney et al., 1988; Moore and Linthicum, 2007). In other words, it is likely that 3-year-olds cannot use auditory fluency because their perceptual system does not provide sufficiently contrasted information about the quality of the signals.

To further investigate this hypothesis, a follow-up experiment was conducted to evaluate children’s reaction times when respectively facing fluent versus dysfluent sentences. As mentioned in our introduction, reaction time is a classical index of fluent processing (e.g., Koch and Forgas, 2012). We hypothesized that 3-year-olds would have the same latency response for fluent and dysfluent sentences, while 4- and 5-year-olds would respond faster to fluent than dysfluent sentences.

## EXPERIMENT 3

### METHOD

#### Participants

This experiment involved 67 children: 22 3-year-olds (11 girls, *M*age = 41.6 months, SD = 3.72, range 36–47 months), 24 4-year-olds (17 girls, *M*age = 54.41 months, SD = 3.39, range 49–59 months), and 21 5-year-olds (8 girls, *M*age = 65.19 months, SD = 3.66, range 60–71 months) from a school in Lyon (France). The demographics were similar to those of Experiments 1 and 2. Each child was tested individually in a quiet room by a single experimenter for about 10 min. Informed parental consent was obtained for each child. All participants were treated according to the Declaration of Helsinki.

### MATERIALS AND PROCEDURE

To test if our dysfluent sentences require longer time latency than our fluent sentences, and to test if this difference emerges from 4 years onward, the following task was used: for each item, children were shown a screen divided into two parts (black and white) picturing two different colored boxes (**Figure 3**). The experimenter said to the children: “In this game, we are going to search for some objects. Each time, only one object will be in one of the two boxes. Each time, a girl will tell you where the object is. If the girl says that the object is in the box placed on the black side, you have to press this button (the experimenter pointed to a black button on the keyboard). If the girl says that the objects is in the box placed on the white side, you have to press this button (the experimenter pointed to a white button on the keyboard). In this game, you have to answer as quickly as possible.” Children were then invited to place their index fingers on the two buttons. Using the same headphones as in Experiments 1 and 2, children then heard for instance, “The ball is in the orange box,” when viewing a blue and an orange box (the sentences of Experiment 1 were used for this experiment). A warm-up (four items: two dysfluent, two fluent) was used to provide feed-back regarding children’s accuracy and the need to respond as quickly as possible. During this warm-up, instructions were repeated if needed. The task was created using the software program E-prime to randomly present the sentences, to record which button was pressed and the reaction time linked to each response (measured in ms). Only reaction times linked to accurate responses were analyzed. After the warm-up, the task *per se* involved eight items: four with fluent sentences and four with dysfluent sentences.

### RESULTS

Before analyzing reaction time results, the accuracy for each kind of sentence was examined. The percentage of accuracy for the fluent sentences was 88.6% for the 3-year-olds, 94.8% for the 4-year-olds, and 90.5% for the 5-year-olds. The percentage of accuracy for the dysfluent sentences was 87.5% for the 3-year-olds, 96.9% for the 4-year-olds, and 94% for the 5-year-olds. There was no significant difference between the accuracy for fluent and dysfluent sentences, both for all children, *t*(66) = -7.53, *p* = 0.454, and for each age group [3-year-olds: *t*(21) = 0.29, *p* = 0.771; 4-year-olds: *t*(23) = -0.81, *p* = 0.426; 5-year-olds: *t*(20) = -0.90, *p* = 0.379]. This result seems to confirm our previous results regarding the comprehension questions: our fluency manipulation did not influence children’s comprehension.

Regarding the reaction time results, a 3-way ANOVA with Age Group (3-year-olds, 4-year-olds, 5-year-olds) and gender (girl, boy) as between-subjects variables and fluency level (fluent, dysfluent) as within-subjects variable was performed for the mean reaction time. The analysis revealed a significant main effect of age group, *F*(1,61) = 17.83, *p* < 0.001, and a significant interaction effect of age group × fluency level, *F*(2,61) = 3.28, *p* = 0.044. Regardless of the fluency level, 3-year-olds took more time to respond (*M* = 3112.69 ms, SD = 720.19) than 4-year-olds (*M* = 2512.70 ms, SD = 360.43) and 5-year-olds (*M* = 2300.73 ms, SD = 243.71). Four-year-olds’ performance was not significantly different from that of the 5-year-olds. Tests of the simple effect of fluency level for each age group showed that 3-year-old children’s reaction time did not differ between fluent (*M* = 3172.20 ms, SD = 776.24) and dysfluent sentences [*M* = 3053.18 ms, SD = 672.38, *F*(1,21) = 0.98, *p* = 0.332]. In contrast, 4-year-olds responded significantly faster to fluent (*M* = 2422.48 ms, SD = 274.02) than to dysfluent sentences [*M* = 2602.92 ms, SD = 416.39, *F*(1,23) = 7.14, *p* = 0.014]. Five-year-olds also responded significantly faster to fluent sentences (*M* = 2254.69 ms, SD = 225.73) than to dysfluent ones [*M* = 2346.78 ms, SD = 257.73, *F*(1,20) = 5.47, *p* = 0.030].

## GENERAL DISCUSSION

The results of the present paper suggest that message fluency influences 4- and 5-year-old children’s endorsement of statements. As in the adult studies reviewed above, fluency seems to play an epistemic role for children over 4 years old. In the absence of other information linked to the message or to the two informants, children are more likely to accept a fluent statement than a dysfluent one.

Unlike older children, 3-year-olds were not sensitive to the difference of fluency between the two voices heard. Our complementary data seem to indicate that the 3-year-olds results could be explained in terms of auditory immaturity. First, the developmental trajectory observed in our Experiments 1 and 2 regarding the use of fluency as an epistemic cue seems not to be linked to theory of mind development. Indeed, no correlation was found between theory of mind scores and fluent choices. One could argue that other developmental changes occurring between 3 and 4 years old could explain our results. For instance, a developmental change has also been observed between 3- and 4-year-olds regarding executive functions, which were related to theory of mind development (e.g., Carlson and Moses, 2001). Nevertheless, our results concerning, respectively, the perceived auditory quality and the reaction time evaluation seem to offer a more economical explanation of the developmental change observed in the present study. In fact, 3-year-olds did not evaluate differently fluent and dysfluent sentences in terms of auditory quality. Moreover, they responded with the same time latency to fluent and dysfluent sentences. In constrast, 4- and 5-year-old children were able to distinguish fluent and dysfluent sentences in terms of auditory quality. They also responded faster to fluent sentences than to dysfluent ones. Thus, these results, together with anatomical data (Kinney et al., 1988; Moore and Linthicum, 2007), offer evidence that 3-year-olds cannot use auditory fluency because their perceptual system does not provide sufficiently contrasted information about the signals’ auditory quality. Interestingly, follow-up analyses showed that 3-year-olds nevertheless used a sort of strategy during the conflicting sources task^3^. In Experiments 1 and 2, they chose significantly more often the last sentence provided by one of the two informants [Experiment 1: 64.2%, *t*(26) = 2.06, *p* = 0.049; Experiment 2: 62.5%, *t*(23) = 2.14, *p* = 0.043]. Because of the counterbalancing, this response strategy led to null results. In contrast, 4- and 5-year-olds were at chance regarding the choice of the last sentence, in Experiment 1 [4-year-olds: 43.6%, *t*(25) = -0.90, *p* = 0.376; 5-year-olds: 57.1%, *t*(27) = 1.21, *p* = 0.237] as well as in Experiment 2 [4-year-olds: 55.5%, *t*(26) = 1.29, *p* = 0.207; 5-year-olds: 50.9%, *t*(26) = 0.25, *p* = 0.802].

Other results of our study could also shed light on what is at stake in 4- and 5-year-olds results. The results of the 4- and 5-year-old children could be interpreted in two ways. According to a *message-based* hypothesis, only the characteristic of the message, here fluency, plays a role in children’s decision. In the literature dealing with the effect of fluency on credibility judgments in adults, it was until now the only dimension taken into account, insofar as the information was not linked to a specific informant [for instance in the Reber and Schwarz (1999), adults had to read sentences on a computer screen]. An *informant-based* hypothesis, in contrast, supposes that children use fluency to attribute characteristics to the informants, such as familiarity or linguistic membership. As mentioned above, recent studies have shown that familiarity and group membership influence testimony selection in young children (e.g., Corriveau and Harris, 2009b; MacDonald et al., 2013). Nevertheless, our results for the preference questions do not support the *informant-based* hypothesis. Children, in each age group, did not express a preference for the character linked to the fluent sentences. Of course, further research is necessary to deepen our understanding of the mechanisms underlying the answers given by the children in our experiments.

One interesting question remains: is it adaptive for children to use auditory fluency as a heuristic to endorse a statement, as they do in our experiment from the age of 4? On the one hand, as mentioned above, adults use the fluency with which an utterance is processed as a basis for their credibility judgments. It has been shown, with a modelisation based on Bayes’ theorem, that fluency is a reliable cue for truth appraisal (Reber and Unkelbach, 2010). Thus, from a general viewpoint, fluency is a valid cue for the trustworthiness of a message (in the absence of malevolent intentions of potential informants). It is therefore arguable, that, when two prima facie equally trustworthy informants offer incompatible advice, differential fluency constitutes an indicator of epistemic reliability.

On the other hand, as mentioned above, processing fluency can be experimentally manipulated. For instance, misleading impressions of truth are created by high contrasts in the visual presentation of a given statement (perceptual fluency), whether it is true or not (Reber and Schwarz, 1999). In real life, however, people are often able to use fluency with an understanding of its actual predictive value, what authors have called the “ecological validity of fluency” (Herzog and Hertwig, 2013).

Nevertheless, all these studies involved adults and further research is needed to better understand how and in what circumstances fluency can play a role in children’s endorsement of statements. These studies should also involve other instantiations of fluency. While we have operationalised fluency via an auditory manipulation, it would be worthwhile to investigate the effects of fluency through other kinds of operationalisation such as repetition, semantic priming and rhyming [see the McGlone and Tofighbakhsh (2000) study on adults], and to test the effects of these different kinds of fluency on children’s testimony selection. In line with the research on adults, we might expect that any variable that increases processing fluency should have the same effect on epistemic decision as our auditory manipulation. The aim of the present study was to test the effect of fluency, as a generic cue, on preschoolers’ endorsement of statements. Even if, as we have just mentioned, any instantiations of fluency would be valid to test its effect, the use of auditory fluency is of particular interest regarding the matter of how speech processing in noise can also modulate the endorsement of information. In adults and children, a large literature has studied speech perception in noise (e.g., Anderson et al., 2010; Song et al., 2012; Strait et al., 2013). Moreover, some studies have tested how a noisy environment influences scholastic achievement (Shield and Dockrell, 2003, 2008). Given that the noise levels in today’s classrooms commonly exceed recommended levels (e.g., American National Standards Institute, 2002; Bradley and Sato, 2008), our study has ecological validity and opens up a possible field of research concerning the matter of how noise may influence, not only speech perception or scholastic achievement, but also the endorsement of spoken utterances.

Finally, the relative weight given to the epistemic cues involved might also be explored by allowing them to conflict. Recently, a study with adults has investigated this kind of conflict by contrasting fluency and accuracy (Henkel and Mattson, 2011). The study showed that the judgment of credibility generated by fluency (here, a repeated exposure to statements) is independent of the reliability of the source. In children, the relationships between different cues, and their relative weights, have also been tested. For instance, Corriveau et al. (2013) have investigated how children respectively weigh accent and accuracy. They showed that endorsements of object labels depend on accuracy rather than accent in 4- and 5-year-old children but not in 3-year-olds—who performed at chance. Further research needs to be carried out on the relations between fluency and other cues, for instance when fluency conflicts with expertise or consensus.

Our results constitute a first attempt to show that fluency is used as an epistemic cue for endorsing statements from age four on. Given the demonstrated importance of fluency for adults, it is important to improve our understanding of the role of fluency in child development. Such research could, for instance, have important pedagogical consequences: on the one hand, some types of knowledge transmission and acquisition are facilitated via fluency. On the other hand, as mentioned above, some misleading impressions of truth are also based on fluency and any ways to increase awareness ot these potentially misleading cues would be beneficial, for children and adults.

## Conflict of Interest Statement

The authors declare that the research was conducted in the absence of any commercial or financial relationships that could be construed as a potential conflict of interest.

## Footnotes

1 All comparisons were calculated according to the Bonferroni procedure.

2 The contrast of interest was -2, 1, 1: the 3-year-olds’ performance (coded -2) is lower than that of the 4-year-olds (coded 1), which is similar to that of the 5-year-olds (coded 1). The orthogonal contrast was 0, -1, 1.

3 We thank an anonymous reviewer for drawing our attention on this point.

## References

[B1] Alter A. L. Oppenheimer D. M. (2009). Uniting the tribes of fluency to form a metacognitive nation. *Pers. Soc. Psychol. Rev.* 13 219–235 10.1177/1088868309341564 19638628

[B2] American National Standards Institute. (2002). Acoustical Performance Criteria, Design Requirements, and Guidelines for Schools (Standard S12. 60–2002).

[B3] Anderson S. Skoe E. Chandrasekaran B. Zecker S. Kraus N. (2010). Brainstem correlates of speech-in-noise perception in children. *Hear. Res.* 270 151–157 10.1016/j.heares.2010.08.001 20708671 PMC2997182

[B4] Baum L. A. Danovitch J. H. Keil F. C. (2008). Children’s sensitivity to circular explanations. *J. Exp. Child Psychol.* 100 146–155 10.1016/j.jecp.2007.10.007 18078950 PMC2413431

[B5] Begg I. M. Anas A. Farinacci S. (1992). Dissociation of processes in belief: source recollection, statement familiarity, and the illusion of truth. *J. Exp. Psychol. Gen.* 121 446–458 10.1037/0096-3445.121.4.446

[B6] Bernard S. Mercier H. Clément F. (2012). The power of well-connected arguments: early sensitivity to the connective because. *J. Exp. Child Psychol.* 111 128–135 10.1016/j.jecp.2011.07.003 21899860

[B7] Besken M. Mulligan N. W. (2013). Perceptual fluency, auditory generation, and metamemory: analyzing the perceptual fluency hypothesis in the auditory modality. *J. Exp. Psychol. Learn. Mem. Cogn.* 40 429–440 10.1037/a0034407 24016138

[B8] Birch S. A. J. Vauthier S. A. Bloom P. (2008). Three- and 4-year-olds spontaneously use others’ past performance to guide their learning. *Cognition* 107 1018–1034 10.1016/j.cognition.2007.12.008 18295193

[B9] Bradley J. S. Sato H. (2008). The intelligibility of speech in elementary school class-rooms. *J. Acoust. Soc. Am.* 123 2078–2086 10.1121/1.2839285 18397015

[B10] Brauer M. McClelland G. (2005). L’utilisation des contrastes dans l’analyse des données: comment tester des hypothèses spécifiques dans la recherche en psychologie? [The use of contrasts in data analysis: how to test specific hypotheses in psychological research?]. *L’Année Psychol.* 105 273–305 10.3406/psy.2005.29696

[B11] Brown A. S. Nix L. A. (1996). Turning lies into truths: referential validation of falsehoods. *J. Exp. Psychol. Learn. Mem. Cogn.* 22 1088–1100 10.1037/0278-7393.22.5.1088

[B12] Carlson S. M. Moses L. J. (2001). Individual differences in inhibitory control and children’s theory of mind. *Child Dev.* 72 1032–1053 10.1111/1467-8624.00333 11480933

[B13] Clément F. (2010). To trust or not to trust? Children’s social epistemology. *Rev. Philos. Psychol.* 1 531–549 10.1007/s13164-010-0022-3

[B14] Clément F. Bernard S. Grandjean D. Sander D. (2013). Emotional expression and vocabulary learning in adults and children. *Cogn. Emot.* 27 539–548 10.1080/02699931.2012.724012 23005583

[B15] Corriveau K. H. Harris P. L. (2009a). Preschoolers continue to trust a more accurate informant 1 week after exposure to accuracy information. *Dev. Sci.* 12 188–193 10.1111/j.1467-7687.2008.00763.x 19120427

[B16] Corriveau K. H. Harris P. L. (2009b). Choosing your informant: weighing familiarity and recent accuracy. *Dev. Sci.* 12 426–437 10.1111/j.1467-7687.2008.00792.x 19371367

[B17] Corriveau K. H. Kinzler K. D. Harris P. L. (2013). Accuracy trumps accent when children learn words. *Dev. Psychol.* 49 470–479 10.1037/a0030604 23231692

[B18] Corriveau K. H. Kurkul K. (2014). “Why does rain fall?”: children prefer to learn from an informant who uses non-circular explanations. *Child Dev.* 85 1827–1835. 24646210 10.1111/cdev.12240

[B19] Dechêne A. Stahl C. Hansen J. Wänke M. (2010). The truth about the truth: a meta-analytic review of the truth effect. *Pers. Soc. Psychol. Rev.* 14 238–257 10.1177/1088868309352251 20023210

[B20] Goldinger S. D. Kleider H. M. Shelley E. (1999). The marriage of perception and memory: creating two-way illusions between words and voices. *Mem. Cogn.* 27 328–338 10.3758/BF03211416 10226442

[B21] Harris P. L. (2012). *Trusting What You’re Told: How Children Learn from Others*. Cambridge, MA: Belknap Press/Harvard University Press 10.4159/harvard.9780674065192

[B22] Hasher L. Goldstein D. Toppino T. (1977). Frequency and the conference of referential validity. *J. Verbal Learn. Verbal Behav.* 16 107–112 10.1016/S0022-5371(77)80012-1

[B23] Henkel L. A. Mattson M. E. (2011). Reading is believing: the truth effect and source credibility. *Conscious. Cogn.* 20 1705–1721 10.1016/j.concog.2011.08.018 21978908

[B24] Herzog S. M. Hertwig R. (2013). “The ecological validity of fluency,” in *The Experience of Thinking: How the Fluency of Mental Processes Influences Cognition and Behavior,* eds Unkelbach C. Greifeneder R. (New York: Psychology Press), 190–219.

[B25] Jenkins J. M. Astington J. W. (1996). Cognitive factors and family structure associated with theory of mind development in young children. *Dev. Psychol.* 32 70–78 10.1037/0012-1649.32.1.70

[B26] Johnston W. A. Dark V. J. Jacoby L. L. (1985). Perceptual fluency and recognition judgments. *J. Exp. Psychol. Learn. Mem. Cogn.* 11 3–11 10.1037/0278-7393.11.1.3 1827829

[B27] Kelley C. M. Rhodes M. G. (2002). Making sense and nonsense of experience: attributions in memory and judgment. *Psychol. Learn. Motiv.* 41 293–320 10.1016/S0079-7421(02)80010-X

[B28] Kinney H. C. Brody B. A. Kloman A. S. Gilles F. H. (1988). Sequence of central nervous system myelination in human infancy. II. Patterns of myelination in autopsied infants. *J. Neuropathol. Exp. Neurol.* 47 217–234 10.1097/00005072-198805000-00003 3367155

[B29] Koch A. S. Forgas J. P. (2012). Feeling good and feeling truth: the interactive effects of mood and processing fluency on truth judgments. *J. Exp. Soc. Psychol.* 48 481–485 10.1016/j.jesp.2011.10.006

[B30] Koenig M. A. Clément F. Harris P. L. (2004). Trust in testimony: children’s use of true and false statements. *Psychol. Sci.* 15 694–698 10.1111/j.0956-7976.2004.00742.x 15447641

[B31] Koenig M. A. Harris P. L. (2005). Preschoolers mistrust ignorant and inaccurate speakers. *Child Dev.* 76 1261–1277 10.1111/j.1467-8624.2005.00849.x 16274439

[B32] Koenig M. A. Jaswal V. K. (2011). Characterizing children’s expectations about expertise and incompetence: halo or pitchfork effects? *Child Dev.* 82 1634–1647 10.1111/j.1467-8624.2011.01618.x 21790541

[B33] Kornell N. Rhodes M. G. Castel A. D. Tauber S. K. (2011). The ease-of-processing heuristic and the stability bias: dissociating memory, memory beliefs, and memory judgments. *Psychol. Sci.* 22 787–794 10.1177/0956797611407929 21551341

[B34] Lucas A. J. Lewis C. Pala F. C. Wong K. Berridge D. (2013). Social-cognitive processes in preschoolers’ selective trust: three cultures compared. *Dev. Psychol.* 49 579–590 10.1037/a0029864 22946437

[B35] MacDonald K. Schug M. Chase E. Barth H. (2013). My people, right or wrong? Minimal group membership disrupts preschoolers’ selective trust. *Cogn. Dev.* 28 247–259 10.1016/j.cogdev.2012.11.001

[B36] McDonough I. M. Gallo D. A. (2012). Illusory expectations can affect retrieval-monitoring accuracy. *J. Exp. Psychol. Learn. Mem. Cogn.* 38 391–404 10.1037/a0025548 21942496

[B37] McGlone M. S. Tofighbakhsh J. (2000). Birds of a feather flock conjointly (?): rhyme as reason in aphorisms. *Psychol. Sci.* 11 424–428 10.1111/1467-9280.00282 11228916

[B38] Mercier H. Bernard S. Clément F. (2014). Early sensitivity to arguments: how preschoolers weigh circular arguments. *J. Exp. Child Psychol.* 125 102–109 10.1016/j.jecp.2013.11.011 24485755

[B39] Moore J. K. Linthicum F. H. (2007). The human auditory system: a timeline of development. *Int. J. Audiol.* 46 460–478 10.1080/14992020701383019 17828663

[B40] Pasquini E. Corriveau K. H. Koenig M. A. Harris P. L. (2007). Preschoolers monitor the relative accuracy of informants. *Dev. Psychol.* 43 1216–1226 10.1037/0012-1649.43.5.1216 17723046

[B41] Perner J. Leekam S. R. Wimmer H. (1987). Three-year-olds’ difficulty with false belief: the case for a conceptual deficit. *Br. J. Dev. Psychol.* 5 125–137 10.1111/j.2044-835X.1987.tb01048.x

[B42] Proust J. (2012). “Metacognition and mindreading: one or two functions?,” in *The Foundations of Metacognition,* eds Beran M. Brandl J. L. Perner J. Proust J. (Oxford: Oxford University Press), 234–251.

[B43] Reber R. Schwarz N. (1999). Effects of perceptual fluency on judgments of truth. *Conscious. Cogn.* 8 338–342 10.1006/ccog.1999.0386 10487787

[B44] Reber R. Unkelbach C. (2010). The epistemic status of processing fluency as source for judgments of truth. *Rev. Philos. Psychol.* 1 563–581 10.1007/s13164-010-0039-7 22558063 PMC3339024

[B45] Rhodes M. G. Castel A. D. (2009). Metacognitive illusions for auditory information: effects on monitoring and control. *Psychon. Bull. Rev.* 16 550–554 10.3758/PBR.16.3.550 19451383

[B46] Schwarz N. (2004). Metacognitive experiences in consumer judgment and decision making. *J. Consum. Psychol.* 14 332–348 10.1207/s15327663jcp1404_2

[B47] Schwarz N. (2010). “Meaning in context: metacognitive experiences,” in *The Mind in Context*, eds Mesquita B. Barrett L. F. Smith E. R. (New York: Guilford Press), 105–125.

[B48] Scofield J. Behrend D. A. (2008). Learning words from reliable and unreliable speakers. *Cogn. Dev.* 23 278–290 10.1016/j.cogdev.2008.01.003

[B49] Shield B. M. Dockrell J. E. (2003). The effects of noise on children at school: a review. *J. Building Acoust.* 10 97–116 10.1260/135101003768965960

[B50] Shield B. M. Dockrell J. E. (2008). The effects of environmental and classroom noise on the academic attainments of primary school children. *J. Acoust. Soc. Am.* 123 133–144 10.1121/1.2812596 18177145

[B51] Sobel D. M. Corriveau K. H. (2010). Children monitor individuals’ expertise for word learning. *Child Dev.* 81 669–679 10.1111/j.1467-8624.2009.01422.x 20438467

[B52] Song J. H. Skoe E. Banai K. Kraus N. (2012). Training to improve hearing speech in noise: biological mechanisms. *Cereb. Cortex* 22 1180–1190 10.1093/cercor/bhr196 21799207 PMC3450924

[B53] Sperber D. Clément F. Heintz C. Mascaro O. Mercier H. Origgi G. (2010). Epistemic vigilance. *Mind Lang.* 24 359–393 10.1111/j.1468-0017.2010.01394.x

[B54] Strait D. L. O’Connell S. Parbery-Clark A. Kraus N. (2013). Biological impact of preschool music classes on processing speech in noise. *Dev. Cogn. Neurosci.* 6 51–60 10.1016/j.dcn.2013.06.003 23872199 PMC3844086

[B55] Weaver K. Garcia S. M. Schwarz N. Miller D. T. (2007). Inferring the popularity of an opinion from its familiarity: a repetitive voice can sound like a chorus. *J. Pers. Soc. Psychol.* 92 821–833 10.1037/0022-3514.92.5.821 17484607

[B56] Wellman H. M. Cross D. Watson J. (2001). Meta-analysis of theory-of-mind development: the truth about false belief. *Child Dev.* 72 655–684 10.1111/1467-8624.00304 11405571

[B57] Wendorf C. A. (2004). Primer on multiple regression coding: common forms and the additional case of repeated contrasts. *Underst. Stat.* 3 47–57 10.1207/s15328031us0301_3

[B58] Whittlesea B. W. A. (2004). The perception of integrality: remembering through the validation of expectation. *J. Exp. Psychol. Learn. Mem. Cogn.* 30 891–908 10.1037/0278-7393.30.4.891 15238031

[B59] Whittlesea B. W. A. Jacoby L. L. Girard K. (1990). Illusions of immediate memory: evidence of an attributional basis for feelings of familiarity and perceptual quality. *J. Mem. Lang.* 29 716–732 10.1016/0749-596X(90)90045-2

[B60] Whittlesea B. W. A. Williams L. D. (1998). Why do strangers feel familiar, but friends don’t? A discrepancy-attribution account of feelings of familiarity. *Acta Psychol.* 98 141–165 10.1016/S0001-6918(97)00040-1 9621828

[B61] Whittlesea B. W. A. Williams L. D. (2000). The source of feelings of familiarity: the discrepancy-attribution hypothesis. *J. Exp. Psychol. Learn. Mem. Cogn.* 26 547–565 10.1037/0278-7393.26.3.547 10855417

[B62] Wimmer H. Perner J. (1983). Beliefs about beliefs: representation and constraining function of wrong beliefs in young children’s understanding of deception. *Cognition* 13 103–128 10.1016/0010-0277(83)90004-5 6681741

[B63] Yue C. L. Castel A. D. Bjork R. A. (2013). When disfluency is—and is not—a desirable difficulty: the influence of typeface clarity on metacognitive judgments and memory. *Mem. Cogn.* 41 229–241 10.3758/s13421-012-0255-8 22976883

